# Thyroid-reproductive axis interplay: immunological mechanisms and implications for female reproductive health

**DOI:** 10.3389/fcimb.2025.1653380

**Published:** 2026-02-02

**Authors:** Sisi Chen, Shahid Ullah Khan, Safir Ullah Khan, Mohammed Alissa, Essam H. Ibrahim, Saleem Ahmad, Ramadan Taha, Kun Zhou

**Affiliations:** 1 Department of Gynecology, Taihe Hospital, Hubei University of Medicine, Shiyan, China; 2 Department of Biomedical Sciences, College of Medicine, Dubai Medical University (COM, DMU), Dubai, United Arab Emirates; 3 Hefei National Laboratory for Physical Science at the Microscale, School of Life Sciences, University of Science and Technology of China, Hefei, China; 4 Department of Medical Laboratory, College of Applied Medical Sciences, Prince Sattam bin Abdulaziz University, Al-Kharj, Saudi Arabia; 5 Biology Department, Faculty of Science, King Khalid University, Abha, Saudi Arabia; 6 Blood Products Quality Control and Research Department, National Organization for Research and Control of Biologicals, Cairo, Egypt; 7 Department of Cell Biology and Physiology, University of Kansas Medical Center, Kansas City, KS, United States; 8 Department of Vascular Surgery, Taihe Hospital, Hubei University of Medicine, Shiyan, China

**Keywords:** thyroid autoimmunity, maternal-fetal interface, inflammatory cytokines, reproductive immunology, placental development

## Abstract

Thyroid dysfunction is a common endocrine disease among women of childbearing age, which seriously affects reproductive health. From an immunological perspective, this in-depth analysis clarifies the complex relationship between thyroid function and female reproduction. We studied the hypothalamic-pituitary-gonadal axis regulation by thyroid hormones through direct and indirect mechanisms, including metabolic mediators such as prolactin and leptin. Recent studies have shown that inflammatory cytokines (IL-1α, IL-1β, IL-6, IFN-γ, and TNF-α) severely disrupt the production pathways of thyroid hormones, establishing an essential link between immune activation and reproductive problems. Since the placenta serves as an active immune interface affected by thyroid activity, there are significant physiological obstacles (including increased iodine clearance and elevated deiodinase activity), immunological challenges (such as altered cytokine profiles), and pathological barriers to optimal thyroid adaptation during pregnancy. This literature review indicates that thyroid problems substantially affect reproductive outcomes by altering the immune response at the maternal-fetal interface, influencing placental development, trophoblast invasion, and vascular remodeling. This review addresses a notable research deficiency through a modern perspective on thyroid dysfunction and reproductive issues, especially inflammatory cytokines related to preeclampsia. We believe that thyroid dysfunction can alter the expression of specific angiogenic factors (including sFlt-1, PlGF, and VEGF) and modify the immune cell profile at the maternal-fetal interface (particularly NK cells, macrophages, and T regulatory cells), creating a new framework for understanding and addressing thyroid-related reproductive diseases through targeted immunomodulatory strategies.

## 1 Introduction

Thyroid disorders affect a substantial share of the population and are considered the most prevalent endocrine condition (Khattak et al., 2016). Before the adoption of iodine fortification across Europe, goitre represented a significant public health concern. Notably, higher iodine intake has been associated with an increased prevalence of hypothyroidism (Ittermann et al., 2015). Approximately 14% of adult women suffer from thyroid diseases, making it one of the most common endocrine disorders among women of childbearing age (Strikić Đula et al., 2022). Studies have shown that women are 3 to 5 times more likely than men to require treatment for thyroid dysfunction (Stoll, 2019; Abdulla et al., 2020). Meta-analytical evidence has shown that specific populations, including individuals with obesity and those of East Asian descent, demonstrate higher susceptibility to thyroid diseases (Song et al., 2019). The clinical significance of these diseases includes several harmful reproductive and metabolic consequences (Dhillon-Smith et al., 2020; Shekhar et al., 2021). For instance, studies have identified the link between subclinical hypothyroidism (SCH) and polycystic ovary syndrome (PCOS) - a reproductive disorder characterized by excessive androgen, irregular ovulation, and unique morphological features of the ovaries (Fatima et al., 2020). In the polycystic ovary syndrome cohort, individuals with SCH showed an increased frequency of metabolic disorders, especially dyslipidemia and decreased insulin sensitivity (de Medeiros et al., 2018). Autoimmune thyroid diseases, such as Graves’ disease and Hashimoto’s thyroiditis, have a terrible impact on reproduction. The prevalence of infertility is close to 50%, and the risk of ovarian insufficiency is relatively high (Quintino-Moro et al., 2014; Hsieh and Ho, 2021).

The process by which thyroid diseases affect female reproductive capacity is complex and functions at multiple levels of the reproductive system (Silva et al., 2018). Thyroid hormones (THs) regulate the production of kisspeptin and gonadotropin-releasing hormone (GnRH) in the hypothalamic-pituitary complex through direct pathways. Additionally, THs influence reproductive function through indirect mechanisms, including metabolic mediators such as prolactin and leptin (Sonigo et al., 2012; Christen et al., 2018; Brown et al., 2019). In addition, thyroid function affects the bioavailability of sex hormones by altering the way binding proteins work. Normal thyroid function is crucial for the normal development and operation of female reproductive organs (Mukku et al., 1983; Wakim et al., 1993; Aghajanova et al., 2011), and is essential for promoting the development of the placenta and the growth of the fetus during pregnancy (Skjöldebrand et al., 1982; Glinoer et al., 1990; Tsuda et al., 2019). Due to the complex interaction between thyroid function and reproductive health, a comprehensive review of the existing information is crucial for strengthening the treatment strategies for individuals with both thyroid and reproductive problems (Hoeger et al., 2021; Weetman, 2021). This review paper integrates recent advances in understanding thyroid-immune interactions, including single-cell RNA sequencing studies and novel insights into decidual immune cell populations, to examine the relationship between the thyroid and the reproductive axis.

## 2 Functional organization of the HPT axis: mechanisms of regulation

**Figure 1:**
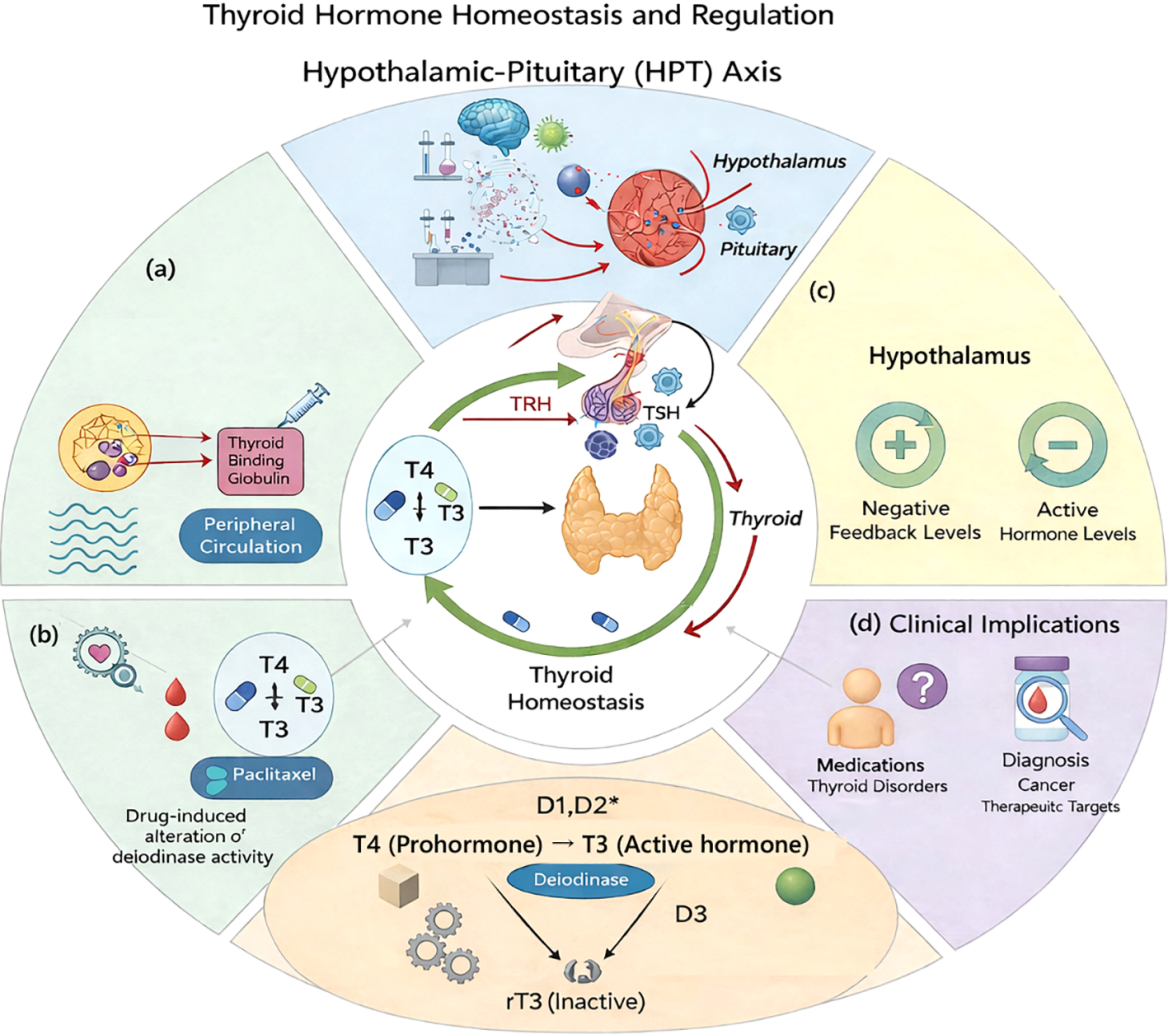
Thyroid Hormone Homeostasis and Regulation. **(a)** Transport and conversion of circulating T4 to bioactive T3 in peripheral tissues. **(b)** Negative feedback of circulating thyroid hormones on TRH and TSH secretion to maintain homeostasis. **(c)** Influence of medications (e.g., paclitaxel) on peripheral T4–T3 conversion pathways. **(d)** Diagnostic and therapeutic applications related to thyroid dysfunction and endocrine disorders. Deiodinase Function: Roles of D1, D2, and D3 in activating (T4→T3) or inactivating (T4→rT3/T3→T2) thyroid hormones.

The hypothalamic-pituitary-thyroid (HPT) axis constitutes a complex regulatory network vital for metabolic regulation and physiological equilibrium. The hypothalamus releases thyrotropin-releasing hormone (TRH) in response to energy-sensing chemical cues, such as leptin, NPY, and AgRP. This starts the functional sequence of the HPT axis (**Figure 1**) (Nillni, 2010).

When TRH is released, it tells the thyrotropic cells in the front of the pituitary gland to make thyroid-stimulating hormone (TSH). TSH then tells the thyroid gland to make triiodothyronine (T3) and tetraiodothyronine (T4). The circulation of these thyroid hormones is mainly facilitated by specialized transport proteins, including thyroxine-binding globulin, transthyretin, and albumin (Braun and Schweizer, 2018). The thyroid gland mostly makes T4, but peripheral tissues include deiodinase enzymes (types 1 and 2) that change T4 into T3, a hormone with more biological activity (Köhrle and Frädrich, 2022). Deiodinase 3, conversely, helps change T3 into reverse T3 (rT3), a physiologically inactive form that acts as a regulatory buffer against too much T3 production (**Figure 1**). This peripheral conversion process is a one-of-a-kind regulatory feature that only applies to thyroid management. Harmful feedback suppression of TRH and TSH release at the hypothalamus and pituitary levels completes the regulatory loop for T3 and T4 (**Figure 1**). Thyroid hormones enter target cells at the cellular level through particular membrane transporters, such as monocarboxylate transporter 8 and 10, or by passive diffusion (Brent, 2012). T3 binds to thyroid receptors inside the cell. When these thyroid receptors are linked to retinoid X receptors (which act as obligate partners for thyroid hormone receptor function) and appropriate coactivators (protein complexes that enhance gene transcription), they modulate gene transcription to mediate thyroid hormone effects.

### 2.1 Changes in the endocrine system during pregnancy and related disorders

Pregnancy causes significant changes in the endocrine system, which can make it harder to diagnose and treat endocrine diseases. Numerous symptoms of normal pregnancy coincide with those of endocrine disorders, and pregnancy itself modifies baseline biochemical parameters (Oude Rengerink et al., 2015). For example, hyperemesis gravidarum causes nausea, vomiting, weakness, drowsiness, irritability, and sadness, which are all symptoms that could hide an underlying endocrine problem. These symptoms happen because there is too much human chorionic gonadotropin (hCG), which makes progesterone and estrogens that are needed to keep a pregnancy going (Velegrakis et al., 2017). As pregnancy continues, the placenta secretes these hormones together with human placental lactogen (hPL), which affects metabolism by boosting the intake of amino acids for fetal development while inducing maternal symptoms such as nausea, headaches, and exhaustion (Nelson-Piercy, 2020).

## 3 Critical windows of inflammatory cytokine expression in pregnancy

IL-1β and TNF-α peaks during implantation (weeks 6-8) coincide with maternal thyroid adaptation requirements. IFN-γ elevation (weeks 14-20) correlates with fetal thyroid gland development. IL-6 surge (weeks 28-36) overlaps with critical fetal brain development periods requiring optimal maternal T4 levels. Each cytokine’s temporal expression pattern is linked to specific thyroid-reproductive consequences with supporting clinical evidence.

### 3.1 Thyroid changes during pregnancy

**Figure 2:**
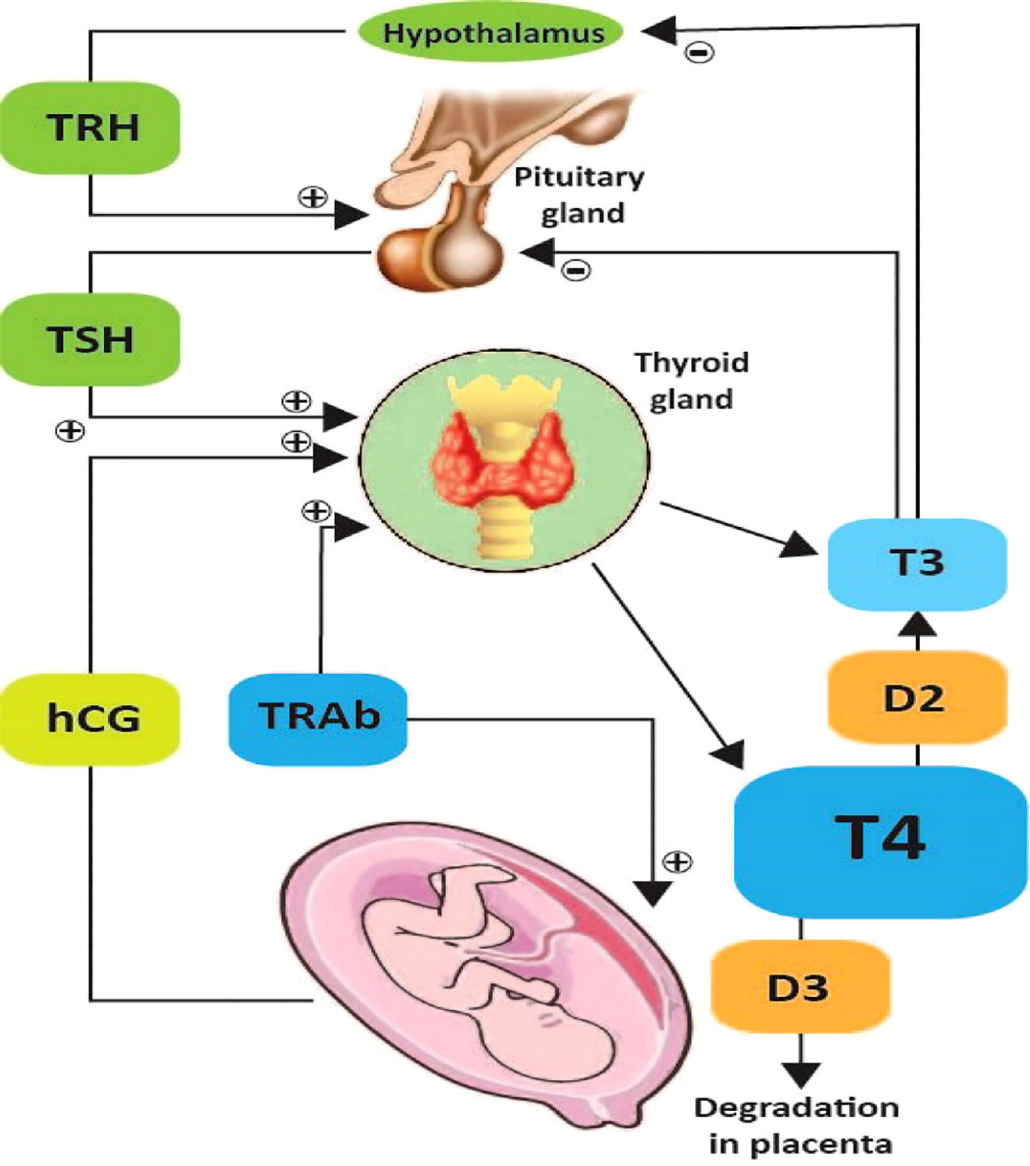
The Hypothalamic-pituitary-thyroid axis during pregnancy. Iodothyronine deiodinase type 3, tetraiodothyronin (T4), triiodothyronine (T3), thyrotropin-releasing hormone (TRH), thyroid-stimulating hormone (TSH), TSH receptor autoantibodies (TRAb), human chorionic gonadotropin (hCG) (Calina et al., 2019).

Pregnancy significantly affects how the thyroid works, mainly because chorionic gonadotropin interacts with the thyroid’s control systems. In the first trimester, hCG strongly stimulates TSH receptors, which improves thyroid function and lowers TSH levels, as shown in **Figure 2**. This establishes an interaction in which rising hCG causes TSH to diminish, which can lead to gestational thyrotoxicosis (Hershman, 2004). When hCG levels drop, TSH levels return to normal after the 12th week. Total thyroxine levels increase beyond non-pregnant reference ranges early in pregnancy due to elevated thyroxine-binding globulin production induced by placental estradiol (Ain et al., 1987). Even with these modifications, free thyroxine usually stays at the top of the non-pregnant reference ranges. Thyroid hormones begin crossing the placenta at modest levels at 6 weeks, emphasizing their essential function in embryonic development (Emerson and Braverman, 1991). The placenta uses type III deiodinase to turn T4 into rT3, which is not physiologically active. This process speeds up in the second part of pregnancy. Maternal TSH cannot traverse the placenta; however, the placenta’s thyrotropin-releasing hormone seems to be produced by the placenta, enhancing fetal thyroid activity by activating the fetal pituitary (Roti et al., 1981).

### 3.2 Hyperthyroidism during pregnancy

About 0.1–0.4% of pregnancies are affected by hyperthyroidism (Lazarus, 2014). Poorly managed hyperthyroidism markedly elevates the risks of pregnancy problems, such as abortion, early birth, preeclampsia, retroplacental hematomas, and maternal heart failure (Sheffield and Cunningham, 2004). Severe uncontrolled hyperthyroidism may cause fetal cardiovascular problems, such as tachycardia, cardiomyopathy, cardiac insufficiency, and hydrops (Männistö et al., 2013). Other problems that can happen to the fetus are stillbirth, acute respiratory distress, and low birth weight for gestational age. The goal of treatment is to get the thyroid to work normally with as little medicine as possible. Antithyroid medications traverse the placenta in negligible quantities, presenting minimal danger of embryonic hypothyroidism. Regular monitoring keeps free thyroxine levels at the upper limit of the reference range. Women with minor thyroid goiters may attain an euthyroid state with little antithyroid medication or by ceasing treatment during late pregnancy (Marino et al., 2006). There are two types of antithyroid drugs. FDA pregnancy category D indicates that there is positive evidence of human fetal risk, but the benefits may warrant use despite potential risks. First, propylthiouracil (PTU), a thiourea derivative designated as FDA category D, inhibits thyroid peroxidase and hinders type I deiodinase, preventing T4 from turning into T3. Second, imidazole derivatives such as methimazole (MMI) and carbimazole (CBZ) block thyroid peroxidase, which lowers the production of T4 and T3. These medicines are the principal therapy for Graves’ illness in pregnancy (Vita et al., 2009). Studies show that MMI and CBZ (FDA category D are linked to aplasia cutis and a rare embryopathy that includes choanal atresia, esophageal problems, and dysmorphological characteristics. Using PTU in the first trimester has been connected to issues with the urinary tract, face, and neck (Bahn et al., 2009).

The Endocrine Society’s current guidelines say that women should convert from MMI/CBZ to PTU during the first trimester and then switch back to MMI/CBZ for the second and third trimesters (De Groot et al., 2012). This method strikes a compromise between dangers because MMI raises the risk of congenital malformations, and PTU raises the risk of maternal hepatotoxicity. The doses of the medication should keep free-T4 levels at the high end of normal for pregnancy. Tests of thyroid function should be done 30 to 40 days after starting medication and then every 4 to 6 weeks. As pregnancy progresses, medication dosages frequently diminish, occasionally permitting cessation. Short-term treatment with propranolol can help with anxiety and palpitations without slowing down fetal growth later.

## 4 The relationship between the thyroid and the female reproductive system

### 4.1 Molecular mechanisms of cytokine-induced thyroid dysfunction

Inflammatory cytokines are secreted through specific signaling pathways: IL-6 binding to its receptor activates JAK1/JAK2, leading to STAT3 phosphorylation and nuclear translocation, where STAT3 recruits transcriptional repressors to the TPO promoter region, directly inhibiting thyroid peroxidase mRNA expression. TNF-α triggers IκB kinase activation, leading to NF-κB nuclear translocation and subsequent inflammatory gene transcription, creating positive feedback loops that amplify thyroid dysfunction. IFN-γ activates p38 MAPK and JNK pathways, resulting in AP-1 transcription factor activation that directly suppresses thyroid-specific genes, including thyroglobulin and sodium-iodide symporter. We explain that inflammation during pregnancy arises from multiple sources: infectious agents triggering pathogen-associated molecular patterns (PAMPs) through Toll-like receptors, metabolic stress from altered glucose and lipid metabolism activating NLRP3 inflammasomes, hormonal fluctuations, particularly estrogen-mediated immune system modifications, and placental hypoxia-reoxygenation cycles generating reactive oxygen species that activate inflammatory cascades, as shown in **Table 1**.

**Table 1:** Cytokine-mediated mechanisms of thyroid dysfunction during pregnancy.

Cytokine	Cellular source	Peak expression window	Molecular mechanism	Thyroid target	Clinical consequence	References
IL-6	Extravillous trophoblasts	Weeks 8-12	JAK1/JAK2→STAT3→HDAC recruitment	TPO gene silencing	Reduced T4/T3 synthesis	(Ajjan et al., 1998)
TNF-α	Decidual macrophages (M1)	Throughout pregnancy	TNFR1→IKK→NF-κB activation	TTF-1/PAX8 interference	Impaired thyroglobulin production	(Vasu et al., 2003)
IFN-γ	Decidual NK cells (70% of leukocytes)	Weeks 14-20	cGAS-STING→STAT1 activation	NIS expression suppression	40% reduction in iodide uptake	(Leon-Letelier et al., 2023)
IL-1β	Peripheral monocytes	Weeks 6-8 (implantation)	NLRP3 inflammasome activation	TSH-induced Tg mRNA	Disrupted hormone synthesis	(Stassi et al., 2000; Fu et al., 2015)
IL-17A	CD4+ Th17 cells	Variable	JAK2/STAT5 pathway inhibition	Progesterone receptor signaling	Pregnancy loss, preeclampsia	(Fu et al., 2015)

When IL-6 inhibits thyroid peroxidase (TPO) mRNA expression, the mechanism involves IL-6 binding to its receptor complex consisting of IL-6 receptor alpha (IL-6Rα) and the signal transducing component gp130. This binding induces conformational changes that bring associated Janus kinases JAK1 and JAK2 into proximity, leading to their activation through transphosphorylation of specific tyrosine residues in their activation loops. Activated JAKs then phosphorylate tyrosine residues (Y767, Y814, Y905, Y915) on the intracellular domain of gp130, creating docking sites for signal transducer and activator of transcription 3 (STAT3) proteins through their SH2 domains. STAT3 undergoes phosphorylation at tyrosine 705 by JAKs, promoting STAT3 dimerization and nuclear translocation. In thyrocytes, phosphorylated STAT3 dimers bind to specific DNA sequences called STAT3 response elements within gene promoters, but paradoxically, instead of activating transcription as in many cell types, STAT3 in thyrocytes recruits transcriptional co-repressors, including histone deacetylases HDAC1 and HDAC3, along with nuclear receptor co-repressor (NCoR), to the TPO gene promoter region. This leads to chromatin condensation through histone deacetylation and transcriptional silencing of TPO expression. For TNF-α effects, we detail how TNF-α binding to tumor necrosis factor receptor 1 (TNFR1) causes receptor trimerization and recruitment of TNF receptor-associated death domain (TRADD) protein to the intracellular domain. TRADD then recruits TNF receptor-associated factor 2 (TRAF2) and receptor-interacting protein kinase 1 (RIPK1), forming a signaling complex that activates the IκB kinase (IKK) complex consisting of IKKα, IKKβ, and IKKγ (NEMO). The IKK complex phosphorylates IκBα proteins at specific serine residues (Ser32 and Ser36), targeting them for K48-linked ubiquitination by the SCF-βTrCP ubiquitin ligase and subsequent proteasomal degradation. This releases nuclear factor-κB (NF-κB) heterodimers (typically p50/p65) from cytoplasmic sequestration, allowing their nuclear translocation where they bind to κB response elements in target gene promoters. In thyrocytes, NF-κB binding to inflammatory gene promoters coincides with interference of thyroid-specific transcription factors thyroid transcription factor-1 (TTF-1) and paired box gene 8 (PAX8) through competitive DNA binding and protein-protein interactions that sequester these factors away from their target promoters.

We have comprehensively mapped the cellular sources and anatomical origins of inflammatory cytokines affecting thyroid-reproductive function with precise quantitative data. At the maternal-fetal interface, decidual natural killer (dNK) cells represent the predominant source of interferon-gamma, constituting approximately 70% of decidual leukocytes and producing 60-70% of total decidual IFN-γ output. These cells display unique phenotypic characteristics (CD56brightCD16-KIR+CD69+) distinct from peripheral NK cells and are strategically positioned around spiral arteries undergoing remodeling. Their IFN-γ production is triggered by trophoblast-derived HLA-G and HLA-E interactions with inhibitory receptors (Gao et al., 2022). Still, pathological conditions can shift them toward excessive IFN-γ secretion, reaching concentrations of 150–200 pg/mL in decidual tissue (compared to <10 pg/mL in maternal serum). Extravillous trophoblasts serve as major sources of IL-6, particularly under conditions of placental hypoxia, where hypoxia-inducible factor-1α (HIF-1α) upregulation drives IL-6 gene transcription. Peak trophoblast IL-6 production occurs during weeks 8–12 of gestation, coinciding with the critical window for maternal thyroid adaptation, with local concentrations reaching 45–60 pg/mL in first-trimester placental tissue. Decidual macrophages, normally polarized toward an alternatively activated (M2) phenotype during healthy pregnancy, can undergo classical (M1) activation under pathological conditions, secreting TNF-α through toll-like receptor 4 (TLR4) pathways activated by oxidized lipids, complement fragments, and damage-associated molecular patterns released from stressed trophoblasts. Systemically, peripheral blood monocytes contribute to IL-1β production through nucleotide-binding oligomerization domain-like receptor protein 3 (NLRP3) inflammasome activation triggered by multiple signals, including thyroid antibody-containing immune complexes, uric acid crystals from altered purine metabolism during pregnancy, and circulating mitochondrial DNA released from placental apoptosis (Zhu et al., 2024). Within thyroid tissue itself, infiltrating immune cells create distinct inflammatory microenvironments: CD4+ T-helper 17 cells produce IL-17A at concentrations of 25–40 pg/mg tissue protein in autoimmune thyroiditis. At the same time, plasma cells secrete not only thyroid-stimulating immunoglobulins but also inflammatory cytokines, including IL-21, that amplify local immune responses (Chang et al., 2016). Visceral adipose tissue, particularly relevant in overweight pregnant patients, contributes to chronic IL-6 production through leptin-mediated activation of resident macrophages and adipocytes, with adipose tissue IL-6 mRNA expression increased 3-5-fold in obese compared to lean pregnant women (Peng et al., 2024).

### 4.2 Neuroendocrine connections: thyroid signaling and regulation of the reproductive axis

The central reproductive system integrates metabolic signals (e.g., glucose, insulin, insulin-like growth factor 1, and leptin), stress-related mediators (e.g., glucocorticoids and corticotropin-releasing hormone), and homeostatic regulators (e.g., thyroid hormones) (Hu et al., 2024). These several signals mostly come together on kisspeptin-neurokinin B-dynorphin neurons. These neurons start signaling along the hypothalamic-pituitary-gonadal (HPG) axis by releasing kisspeptin, which causes specialized hypothalamic neurons to release GnRH in bursts (Navarro, 2020). GnRH neurons incorporate supplementary inputs. After GnRH is produced, the anterior pituitary gonadotrophs release luteinizing hormone (LH) and follicle-stimulating hormone (FSH). These hormones work on theca and granulosa cells in the ovaries to boost the production of gonadal steroids and the growth of follicles.

TRH is the main thing that controls how the HPT axis works, affecting how the HPG axis works. In considerable primary hypothyroidism (Sharma et al., 2016), increased TRH secretion can indirectly influence GnRH release through its substantial stimulatory impact on prolactin synthesis, with prolactin subsequently inhibiting hypothalamic GnRH release. Prolactin is mainly known for its involvement in breast growth and breastfeeding, but it also stops the hypothalamus from releasing GnRH. The mechanisms that cause hyperprolactinemia, affecting the central HPG components, work through many channels. Prolactin directly influences GnRH neurons while concurrently diminishing Kiss1 mRNA expression, limiting subsequent GnRH production (Koike et al., 1991). The rise in prolactin that comes with hypothyroidism also raises dopamine, which is the natural prolactin inhibitor. Changing kisspeptin signaling directly lowers GnRH secretion (Goodman et al., 2012; Liu and Herbison, 2013). Dopamine and prolactin also change how the pituitary responds to GnRH stimulation (Dairaghi et al., 2022). Additionally, prolactin stimulates the release of progesterone from the corpus luteum, which may prolong its functional lifespan (Hapon et al., 2007).

## 5 Thyroid-associated hormones in development

Gestational thyroid function experiences substantial modifications due to pregnancy-induced physiological and metabolic alterations. During early pregnancy, the fetus depends almost exclusively on maternal thyroid hormone provision, necessitating increased maternal thyroid hormone demand and consumption. Key factors include enhanced renal iodine clearance, elevated placental thyroid hormone deiodination, and estrogen-mediated increases in thyroid-binding globulin with corresponding decreases in free thyroid hormones. Early gestational hCG elevation, sharing structural α subunit homology with TSH, exerts mild thyroid-stimulating effects, physiologically elevating maternal thyroid hormone levels while suppressing TSH (Geno and Nerenz, 2022). Some susceptible individuals may inadequately adapt to these physiological changes, developing thyroid dysfunction and increased thyroid disease susceptibility. Maternal gestational thyroid disorders predispose to various pregnancy complications and adverse maternal-fetal outcomes, including preeclampsia, pregnancy loss, and preterm delivery (Korevaar et al., 2017a). Both TSH and thyroid hormones demonstrate significant associations with preeclampsia occurrence and progression.

### 5.1 Infectious etiologies of thyroid-reproductive dysfunction

Beyond SARS-CoV-2, Cytomegalovirus (CMV) infection, present in 0.5-2% of pregnancies, triggers robust IL-6 and TNF-α production from infected trophoblasts through viral protein US2-mediated activation of NF-κB pathways, leading to disrupted placental thyroid hormone transporters (MCT8 and MCT10) and resulting in fetal hypothyroxinemia despite normal maternal thyroid function (Chen et al., 2015). Herpes simplex virus (HSV) infection induces massive IFN-γ secretion from decidual NK cells through viral DNA recognition by cGAS-STING pathways, directly inhibiting maternal sodium-iodide symporter (NIS) expression through STAT1-mediated transcriptional suppression, reducing thyroidal iodide uptake by up to 40%. Epstein-Barr virus (EBV) demonstrates molecular mimicry with thyroid peroxidase, where viral glycoproteins share epitopes with TPO, leading to cross-reactive antibody production and autoimmune thyroiditis development in 15% of infected pregnant women (Figliozzi et al., 2017). Zika virus crosses the placental barrier and directly infects fetal thyroid tissue, triggering neuroinflammation with IL-1β levels exceeding 50 pg/mL (normal <5 pg/mL), compounding neurodevelopmental risks in fetuses already compromised by maternal hypothyroxinemia. Bacterial pathogens present distinct mechanisms: Chlamydia trachomatis chronic infection maintains elevated systemic TNF-α levels (>20 pg/mL) through persistent antigen presentation via MHC-II complexes, correlating with 3-fold increased anti-TPO antibody titers (Elgueta et al., 2022). Ureaplasma urealyticum intrauterine infection triggers potent IL-8 and IL-1β production through MyD88-dependent TLR signaling, disrupting fetal thyroid hormone receptor expression and sensitivity; Group B Streptococcus maternal colonization activates complement cascades through bacterial capsular polysaccharides, leading to immune complex deposition in thyroid tissue and subsequent postpartum thyroiditis in 25% of colonized women (Glaser et al., 2019). We detail how each pathogen leads to specific pregnancy complications, including preterm labor, intrauterine growth restriction, and preeclampsia, through thyroid-mediated mechanisms.

### 5.2 Thyroid hormone associations: thyroid stimulation

Thyroid-stimulating components encompass thyroxine (T4) and the more biologically active triiodothyronine (T3). Approximately 10% of circulating T4 and T3 exist in unbound forms, designated free thyroxine (FT4) and free triiodothyronine (FT3). T3 mediates essential human physiological functions through thyroid nuclear receptor binding, regulating transcriptional processes governing metabolism, growth, and development (Deng et al., 2022). Cohort investigations demonstrate U-shaped associations between FT4 levels and preeclampsia risk (Su et al., 2021), while meta-analytical evidence indicates similar U-shaped correlations between FT3 and gestational hypertension/preeclampsia. Concurrently, elevated total T3 concentrations are associated with gestational hypertension (Derakhshan et al., 2024). Thyroid hormone influences on preeclampsia pathogenesis may relate to placental formation effects and vascular endothelial dysfunction, with elevated anti-angiogenic markers in preeclamptic patients potentially impacting maternal thyroid function.

Mounting evidence reveals abnormal thyroid hormone signaling in placental-mediated pregnancy complications, including preeclampsia, pregnancy loss, and intrauterine growth restriction (IUGR) (Adu-Gyamfi et al., 2020). Thyroid hormones directly target thyroid hormone-specific nuclear receptors on human villous placental tissue, participating in trophoblast proliferation, differentiation, and invasion processes. The investigations demonstrated T3’s role in maintaining stem cell growth while significantly enhancing trophoblast differentiation. Impaired trophoblast invasion and differentiation affect maternal uterine spiral artery remodeling, influencing maternal-fetal interface blood supply and promoting placental hypoxia. Pregnancy complications, including preeclampsia, pregnancy loss, and IUGR, all exhibit these placental alterations. Inadequate maternal-fetal interface perfusion, causing persistent placental oxidative stress, represents a critical preeclampsia onset mechanism (Mallawa Kankanamalage et al., 2021). Research demonstrates that hypothyroid rat maternal-fetal interfaces experience hypoxic conditions with activated oxidative and endoplasmic reticulum stress, further compromising placental growth and development (dos Anjos Cordeiro et al., 2022). Optimal placental development requires proper decidualization, with inadequate decidualization associated with preeclampsia and recurrent pregnancy loss. Maternal-fetal interface natural killer (NK) cells participate in placental development, trophoblast invasion, and vascular remodeling. Hyperthyroid rats exhibit decreased NK cell numbers and increased inflammatory cytokinesexpression (including interferon-γ and interleukin-5), impairing decidualization (Santos et al., 2023). Critical placental formation processes, including trophoblast proliferation and differentiation, decidual cell invasion and angiogenesis, and pro-inflammatory/anti-inflammatory factor regulation, all experience thyroid hormone influence. Maternal thyroid hormone deficiency or excess adversely affects placental development, potentially representing preeclampsia’s primary pathological foundation.

Preeclamptic patients demonstrate angiogenic imbalances characterized by increased peripheral blood soluble fms-like tyrosine kinase-1 (sFlt-1) and soluble endoglin (sEng) concentrations alongside decreased PlGF and VEGF expression, resulting in endothelial dysfunction. PlGF and VEGF represent trophoblast-secreted angiogenic factors promoting early gestational placental angiogenesis while inducing trophoblast growth, differentiation, and invasion. Evidence indicates that the thyroid hormone affects angiogenic factor expression. They demonstrated elevated maternal blood cadmium content in preeclamptic patients, with gestational cadmium exposure increasing preeclampsia risk (Li et al., 2022). Human choriocarcinoma cell cadmium chloride treatment reduces thyroid hormone-specific nuclear receptor and deiodinase type II (Dio2) expression while decreasing PlGF and VEGF expression and increasing sFlt-1 expression. Dio2 represents a critical intracellular T3 concentration regulatory factor affecting cellular thyroid hormone signal transduction. Direct Dio2 inhibitor treatment similarly reduces PlGF expression while increasing sFlt-1 expression, indicating that thyroid hormone receptor signal disorders may cause angiogenic factor imbalances. They identified relatively low Kisspeptin expression levels in preeclamptic patients (Santos et al., 2022). Hypothyroid rat treatment with Kisspeptin improved placental development and plasma FT3 and FT4 levels, potentially relating to increased PlGF and antioxidant enzyme expression alongside positive VEGF regulation. Given thyroid tissue’s high vascularization, elevated sFlt-1 in preeclamptic patients can also impair thyroid angiogenesis. Therefore, sFlt-1 and PlGF are considered potential maternal thyroid function regulatory factors. The experimental findings showed preeclamptic rat thyroid follicular cell destruction, interfering with Dio1 expression, which catalyzes T4 to T3 conversion (Liu et al., 2021). Amlodipine besylate treatment enhances VEGF signaling pathways and improves thyroid follicle and Dio1 expression. In conclusion, abnormal thyroid hormone signaling may influence angiogenic factor expression, while elevated anti-angiogenic factor levels in preeclamptic patients can affect thyroid hormone synthesis and secretion (Kang et al., 2024).

### 5.3 TSH correlations: thyroid-stimulating hormone analysis

Thyroid-stimulating hormone represents the most sensitive physiological marker reflecting maternal thyroid function. Both elevated and decreased TSH levels can increase preeclampsia risk, potentially relating to vasoconstriction function effects and oxidative environment alterations. Researchers’ case-control investigation found significantly increased pulse wave velocity (PWV), measuring arterial stiffness, in preeclamptic patients, with elevated TSH increasing the risk of elevated PWV (Businge et al., 2021b). The underlying mechanism may involve TSH level increases inhibiting endothelial nitric oxide synthase (eNOS), resulting in decreased nitric oxide (NO) levels, impaired vasodilation function, and increased sympathetic nervous tension. Enhanced vascular resistance ultimately leads to hypertension. NO represents a potent vasodilator regulating angiogenic factor expression, with eNOS expression under VEGF regulation. Research demonstrates significantly decreased nitrogen oxide (NOx) concentrations during preeclampsia development. Reduced NO production constitutes a preeclampsia onset risk factor, with NO decreases likely representing both consequences and mediators of systemic endothelial dysfunction (Socha et al., 2022).

Pregnancy-specific reference ranges should be established. The American Thyroid Association (ATA) stipulates that TSH reference ranges from 7–12 gestational weeks can be reduced by approximately 0.4 mIU/L compared to non-pregnant lower limits and by approximately 0.5 mIU/L compared to upper limits (Alexander et al., 2017). Without specific reference ranges, TSH > 4.0 mIU/L can serve as first-trimester upper limit values, with second- and third-trimester pregnancy reference ranges adopted accordingly (Alexander et al., 2017). Pre-pregnancy and gestational thyroid function monitoring and intervention are essential for preeclampsia risk assessment: gestational thyroid disease and preeclampsia correlations. The hypothalamus, pituitary, and peripheral thyroid hormone metabolism pathways all suffer synchronous modifications in non-thyroidal sickness syndrome (NTIS), according to research undertaken on the illness in animal models (Boelen et al., 2004). This means that NTIS encompasses dysfunction in numerous organ systems in addition to central downregulation of the hypothalamic-pituitary-thyroid (HPT) axis. There is evidence that inflammatory cytokines can influence multiple portions of thyroid hormone production pathways, decreasing T4 and T3 secretion, alone or in combination (Bartalena et al., 1998). These inflammatory cytokines well-established inhibitory effects have been discovered in IL-1α, IL-1β, IL-6, IFN-γ, and TNF-α.

### 5.4 Thyroid function disruption mediated by cytokines

**Figure 3:**
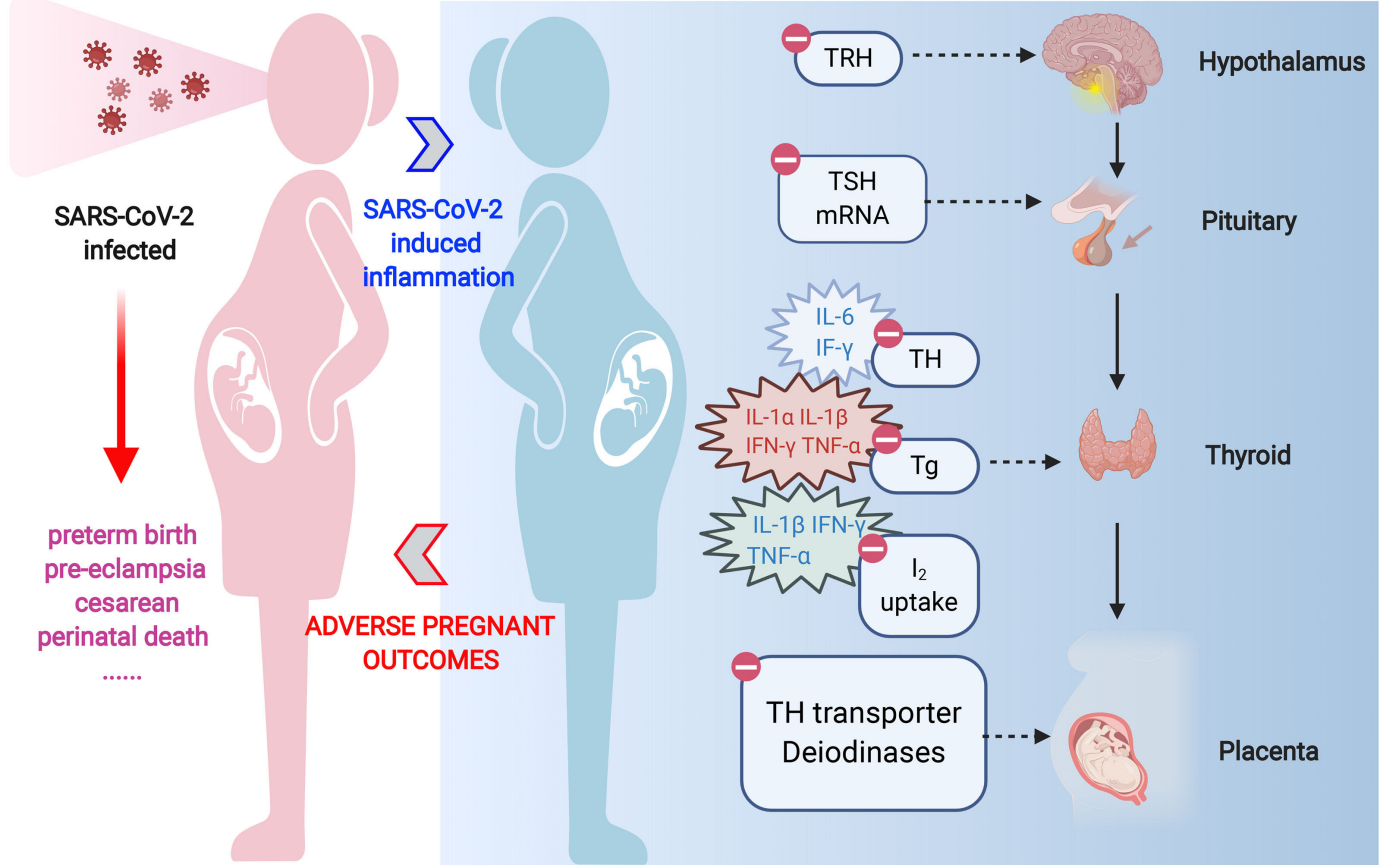
Impact of SARS-CoV-2 infection on thyroid function and pregnancy outcomes (Shi et al., 2022).

Research suggests that thyroglobulin (Tg) gene expression and subsequent protein release from human thyroid cells are disturbed by interleukin-1 variants (IL-1α and IL-1β) when TSH is present (Rasmussen et al., 1988). Moreover, in pig thyroid follicular cells, IL-1β inhibits baseline and TSH-stimulated iodide absorption via the sodium/iodide symporter (Nolte et al., 1994). Clinical investigations show that hospitalized patients’ serum T3 concentrations and IL-6 levels are inversely associated. The ability of IL-6 to decrease thyroid peroxidase (TPO) gene expression and T3 secretion generated by both TSH and cyclic AMP appears to be a significant component in this connection, as shown in **Figure 3**. Furthermore, IL-6 promotes oxidative stress, pointing to a single mechanism in which cytokines’ oxidative damage subsequently changes deiodinase’s expression and activity (Marsili et al., 2011).

Thyroid tissue is affected by interferon-gamma (IFN-γ), which is primarily involved in antimicrobial defense. It decreases Tg mRNA expression and TSH-induced thyroid hormone and thyroglobulin secretion (Nagayama et al., 1987). Additionally, IFN-γ blocks TSH from triggering the synthesis of thyroid peroxidase and disturbs the upregulation of TSH receptors on thyroid cells mediated by TSH and cAMP (Ashizawa et al., 1989). IFN-γ has been demonstrated to block TSH-induced sodium/iodide symporter expression in rat FRTL-5 cell cultures, which lowers iodide uptake and consequent hormone synthesis. TSH-induced cAMP signaling and thyroglobulin synthesis and release in thyroid cell cultures are suppressed by tumor necrosis factor-alpha (TNF-α) (Poth et al., 1991). TNF-α further jeopardizes the basic iodide transport required for thyroid hormone synthesis by reducing sodium/iodide symporter expression in rat thyroid cells, precisely like IFN-γ does (Ajjan et al., 1998).

## 6 Gestational thyroid disorders and their connection to preeclampsia

### 6.1 Gestational thyroid insufficiency: the predominant thyroid abnormality in pregnancy

Among thyroid disorders in pregnancy, hypothyroidism represents the most prevalent condition, with incidence rates approximating 0.3-0.5%. Clinical or overt hypothyroidism (OH) is characterized by decreased FT4 concentrations accompanied by elevated TSH levels; alternatively, TSH values exceeding 10.0 mIU/L warrant OH diagnosis regardless of FT4 status. Subclinical hypothyroidism (SCH) presents when serum TSH exceeds pregnancy-specific reference ranges while FT4 remains within normal parameters. OH, patients demonstrate reduced serum thyroid hormone concentrations, resulting in compromised placental anti-inflammatory environments and vascular abnormalities that contribute to preeclampsia development. Conversely, SCH-associated TSH elevations can increase vascular resistance and promote endothelial dysfunction. The investigation revealed that early pregnancy women with negative thyroid antibodies and TSH values between 4–10 mIU/L demonstrated independent associations between TSH levels and elevated uterine artery pulsatility index (PI) alongside increased preeclampsia risk, as shown in **Table 2**. Numerous cohort investigations have established strong associations between OH, SCH, and preeclampsia susceptibility (Magri et al., 2023).

**Table 2:** Pregnancy-specific thyroid reference ranges and clinical management guidelines.

Parameter	Non-pregnant range	First trimester	Second trimester	Third trimester	Clinical action	References
TSH (mIU/L)	0.4-4.0	0.1-2.5	0.2-3.0	0.3-3.0	Monitor q4–6 weeks if >2.5	(Korevaar et al., 2017a)
Free T4 (pmol/L)	Dec-22	Dec-19	Sep-16	Aug-15	Supplement if <10th percentile	(Dashe et al., 2005)
Free T3 (pmol/L)	3.1-6.8	3.1-4.2	2.6-4.8	2.3-4.2	U-shaped risk for preeclampsia	(Negro et al., 2006)
TPO Antibodies	<35 IU/mL	Same	Same	Same	Monitor TSH monthly if positive	(Negro et al., 2006)
hCG (IU/L)	<5	25,700-288,000	8,900-55,000	940-60,000	Consider GTT if very high with low TSH	(Korevaar et al., 2017b)

### 6.2 Iodine insufficiency and preeclampsia: examining the connection

Iodine deficiency constitutes the primary hypothyroidism etiology, with pregnancy-associated increases in renal iodine clearance and fetal iodine requirements potentially exacerbating maternal iodine insufficiency (Businge et al., 2022). Despite established associations between gestational hypothyroidism and preeclampsia risk, relationships between iodine deficiency and preeclampsia remain incompletely characterized. Meta-analytical evidence demonstrates significantly lower urinary iodine concentrations in preeclamptic women compared to normotensive counterparts (Businge et al., 2021a). These findings revealed that both iodine deficiency severity and oxidized low-density lipoprotein levels, suggesting endothelial dysfunction, increased proportionally with preeclampsia severity, potentially relating to iodine deficiency-induced antioxidant status reductions. Conversely, Finnish case-control research demonstrated no associations between early pregnancy (10–14 weeks) serum iodine concentrations or thyroid function markers and preeclampsia risk. However, thyroid disorder prevalence was significantly higher among preeclamptic patients than non-preeclamptic controls (Reische et al., 2021). Current evidence, therefore, remains insufficient to conclusively establish significant associations between maternal iodine deficiency and preeclampsia development.

### 6.3 Maternal isolated low thyroxine syndrome and preeclampsia risk assessment

Isolated hypothyroxinemia (IH), a condition characterized by normal TSH values alongside reduced FT4 levels, presents with FT4 levels 2.5-5% below reference ranges, with incidence rates approximating 8-10%. Iodine deficiency represents one potential IH etiology. Retrospective cohort analysis revealed a 1.16-fold increased preeclampsia risk among IH patients compared to euthyroid pregnant women. The investigation demonstrated 2.66-fold elevated preeclampsia risk in IH-affected pregnancies compared to normal thyroid function pregnancies, though levothyroxine (LT4) intervention failed to reduce the occurrence risk (Li et al., 2022). Other meta-analyses show no significant differences in gestational hypertension or preeclampsia risk between IH patients and euthyroid controls. Limited and conflicting IH-adverse pregnancy outcome studies exist, potentially relating to inconsistent IH diagnostic criteria across investigations. The American Thyroid Association discourages LT4 treatment for IH-affected pregnancies, as excessive LT4 administration may elevate FT4 levels, which, even subclinically, may increase preeclampsia risk.

### 6.4 Maternal thyroid hyperactivity: impact on pregnancy outcomes

Graves’ disease represents the predominant gestational hyperthyroidism etiology, with incidence rates ranging from 0.5-1.3%. This condition involves TSH receptor stimulation by autoantibodies, resulting in excessive TSH-independent T3 and T4 production. Subclinical hyperthyroidism (SHG) presents with decreased TSH alongside normal thyroid hormone levels. Gestational transient thyrotoxicosis (GTT) represents a temporary thyroid function elevation resulting from high hCG concentrations stimulating TSH receptors, typically resolving by 16 gestational weeks. Inadequately managed hyperthyroidism constitutes a recognized preeclampsia risk factor; antithyroid medication treatment significantly reduces preeclampsia risk, though it remains elevated compared to euthyroid pregnancies (Alves Junior et al., 2022). Pregnancy-associated SHG primarily relates to hCG influences. Research demonstrated 3.4- 4.9-fold increased preeclampsia risk among women with low hCG/high FT4 combinations compared to euthyroid controls, while high hCG/high FT4 combinations demonstrated no elevated preeclampsia risk (Korevaar et al., 2016). Chinese cohort evidence suggests Graves’ disease may increase gestational hypertension risk. At the same time, GTT demonstrates no significant gestational hypertension effects (Xu et al., 2023), potentially indicating that transient hCG-induced GTT rarely causes adverse pregnancy outcomes. Dutch cohort investigations revealed positive linear correlations between FT4 and second/third trimester umbilical artery PI and second trimester uterine artery resistance index, suggesting gestational hyperthyroidism may impact fetal development through increased placental vascular resistance persisting into late pregnancy.

### 6.5 Thyroid autoimmunity in pregnancy: clinical implications

Autoimmune thyroid disease (AITD) presents with positive thyroid autoantibodies thyroid peroxidase autoantibody (TPOAb) or thyroglobulin antibody (TgAb). This condition affects 2.0-17% of pregnant women, with variation depending on geographical iodine status, detection methods for thyroid antibodies, and population characteristics (Fröhlich and Wahl, 2017). TPOAb-positive women demonstrate impaired thyroid function responses to hCG stimulation, potentially reducing serum thyroid hormone levels. Though numerous studies indicate no associations between thyroid autoantibody status and preeclampsia risk, with meta-analytical evidence showing no preeclampsia risk reduction following LT4 treatment in euthyroid TPOAb-positive patients, TPOAb positivity remains the most significant gestational thyroid dysfunction risk factor (Di Girolamo et al., 2022). Furthermore, positive TPOAb status may increase the risks of miscarriage, preterm birth, placental abruption, and preeclampsia in hypothyroid patients. Consequently, antibody monitoring merits emphasis, with recommendations for TSH evaluation every four weeks until the second trimester for women with TSH > 2.5 mIU/L.

Case of a 29-year-old nulliparous woman presenting with recurrent first-trimester losses and subclinical hypothyroidism (TSH 3.8 mIU/L, FT4 12.5 pmol/L, reference 12-22). Detailed immunological profiling revealed elevated IL-17A (38 pg/mL, regular <15), reduced IL-10 (2.1 pg/mL, normal >8), and positive anti-TPO antibodies (280 IU/mL). The critical analysis reveals that IL-17A directly interferes with progesterone receptor signaling through JAK2/STAT5 pathway inhibition, where IL-17 binding to its receptor complex activates TRAF6 and subsequent NF-κB signaling that upregulates suppressor of cytokine signaling 3 (SOCS3), which then binds to and inactivates JAK2 kinase activity essential for progesterone-induced decidualization. This hormonal imbalance manifests as inadequate endometrial preparation, insufficient trophoblast invasion, and eventual pregnancy loss. Therapeutic intervention involved selenium supplementation (200 μg daily selenomethionine), which reduced anti-TPO titers by 60% over 12 weeks through enhanced glutathione peroxidase activity, protecting thyrocytes from oxidative damage, combined with omega-3 fatty acids (2g EPA/DHA daily) that reduced IL-17A levels by 45% through peroxisome proliferator-activated receptor-gamma activation, inhibiting NF-κB-mediated inflammatory gene transcription. Another illustrative case involves a 34-year-old woman with Hashimoto’s thyroiditis developing severe preeclampsia at 28 weeks of gestation (Wu et al., 2023). Critical analysis revealed that chronic thyroid inflammation had triggered a cascade of hormonal imbalances: elevated cortisol (850 nmol/L, normal 200-600) due to hypothalamic-pituitary-adrenal axis activation by persistent IL-1β signaling; disrupted leptin sensitivity (leptin 45 ng/mL with elevated soluble leptin receptor indicating leptin resistance) affecting hypothalamic GnRH pulsatility through STAT3 pathway interference; and altered insulin-like growth factor-1 levels (180 μg/L, normal 250-400) due to inflammatory cytokine inhibition of hepatic IGF-1 synthesis through STAT5 pathway disruption. The consequences included inadequate spiral artery remodeling, placental insufficiency, and progression to severe preeclampsia requiring preterm delivery. Future therapeutic strategies based on this analysis include personalized anti-inflammatory approaches using pregnancy-safe agents like low-dose aspirin combined with antioxidants, targeted cytokine blockade using IL-17A antagonists currently in development, and precision medicine approaches using individual cytokine profiles to guide levothyroxine dosing and monitoring frequency.

## 7 Limitations

The current evidence base suffers from several critical limitations that affect the strength of conclusions we can draw about thyroid-reproductive-immune interactions. Most mechanistic data derive from animal models, particularly rodent studies, which may not accurately reflect human physiology due to species differences in thyroid hormone metabolism, immune system organization, and placental structure. For example, rodent placentation involves different degrees of maternal vascular remodeling compared to human hemochorial placentation. Human studies are predominantly observational and cross-sectional rather than interventional and longitudinal, limiting our ability to establish causality versus correlation between inflammatory markers and thyroid dysfunction. Sample size calculations reveal that most published studies are underpowered to detect clinically meaningful effect sizes, with average study populations of 50–200 subjects when power analyses suggest 400–800 subjects would be needed to detect 20% differences in cytokine levels between groups. Cytokine measurement methodology varies significantly across studies, with some using single-time-point measurements. In contrast, others employ serial sampling, some measuring total cytokine levels while others focus on bioactive forms, and significant inter-laboratory variability in assay standardization affects reproducibility. Confounding variable control remains inadequate across most studies, with insufficient adjustment for maternal age, body mass index, smoking status, concurrent medications, gestational age at sampling, and previous pregnancy outcomes - factors that independently influence both immune function and thyroid status. Publication bias toward positive associations may overestimate the true magnitude of relationships between inflammatory markers and thyroid dysfunction, as suggested by funnel plot asymmetry in recent meta-analyses.

Critical knowledge gaps requiring urgent investigation include: the lack of standardized reference ranges for inflammatory markers during pregnancy, with current “normal” values often derived from non-pregnant populations; limited understanding of epigenetic modifications induced by maternal inflammatory cytokines on fetal thyroid gene expression and long-term offspring thyroid function; insufficient data on the temporal relationship between cytokine elevation and thyroid dysfunction onset; absence of validated biomarkers for early detection of thyroid-immune dysregulation before clinical manifestation; and incomplete characterization of the heterogeneity in individual inflammatory responses to similar stimuli. Future research must prioritize prospective cohort studies with serial sampling from preconception through postpartum periods, standardization of cytokine measurement protocols across research centers, development of predictive models incorporating multiple biomarkers and clinical variables, and investigation of therapeutic interventions targeting specific inflammatory pathways while preserving essential immune functions during pregnancy.

## 8 Future directions and clinical applications

The relationship among thyroid function, immune control, and reproductive health offers a promising approach for future research and clinical progress. Promising strategies should be adopted to enhance our understanding and improve the prognosis of patients. Initially, prospective longitudinal studies should examine the temporal association between thyroid antibody production, specific immune cell populations (particularly decidual NK cells, macrophages, and T regulatory cells), and key cytokines (including IL-10, TNF-α, and IFN-γ) in relation to reproductive outcomes. This research will determine if thyroid autoimmunity precedes immunological alterations at the mother-fetus interaction or the reverse. These investigations must encompass comprehensive evaluations of the cytokine network and immune cell populations in the decidua, correlating them with thyroid measurements during pregnancy. Secondly, complex immune typing methods, such as single-cell RNA sequencing and extensive cytology, should be used to describe the complex cellular relationship between thyroid dysfunction and individual immune cells. These methods can identify new immune cell subsets and signaling pathways related to reproductive problems associated with the thyroid gland, and may discover new therapeutic targets. Thirdly, animal models that selectively target and eliminate thyroid hormone receptors in specific immune cell populations can clarify the direct impact of thyroid hormones on the immune function of reproductive tissues. This model will explain the direct impact of the thyroid gland on immune cells, rather than the indirect impact mediated by metabolic changes. Clinically, these insights have suggested numerous applications. Defining a precise reference range for thyroid measures during pregnancy and accounting for the immune activation state can enhance the accuracy of pregnancy diagnosis. The current reference range utilized by doctors may inadequately represent the physiological alterations that transpire during pregnancy, particularly regarding immunological activation.

Moreover, future research into tailored levothyroxine supplementation strategies informed by individual immunological profiles represents a promising area for investigation. Preliminary evidence suggests that patients with heightened pro-inflammatory cytokines may require different therapeutic approaches compared to those with predominantly anti-inflammatory profiles, though this requires validation through clinical trials. The promise of immunomodulatory treatments for addressing thyroid-related reproductive disorders remains largely unexamined. Targeted intervention to balance pro-inflammatory and anti-inflammatory cytokines or regulate specific immune cell populations at the maternal-fetal interface can enhance traditional thyroid hormone replacement therapy. The pre-pregnancy thyroid screening regimen should be reassessed to incorporate immune function markers alongside conventional thyroid measures. This thorough method can identify women at elevated risk of difficulties before pregnancy, thereby facilitating the implementation of preventive management strategies. Integrating thyroid care with reproductive immunology may enable doctors to devise more effective ways for preventing and treating reproductive problems in women with thyroid dysfunction, hence enhancing maternal and newborn outcomes throughout the reproductive continuum.
